# Efficacy of virtual reality-based intervention on balance and mobility disorders post-stroke: a scoping review

**DOI:** 10.1186/s12984-015-0035-3

**Published:** 2015-05-10

**Authors:** Anuja Darekar, Bradford J McFadyen, Anouk Lamontagne, Joyce Fung

**Affiliations:** School of Physical and Occupational Therapy, Faculty of Medicine, McGill University, Montreal, Quebec Canada; Feil and Oberfeld Research Center, Jewish Rehabilitation Hospital, Research site of the Montreal Centre for Interdisciplinary Research in Rehabilitation (CRIR), Laval, Quebec Canada; Centre for Interdisciplinary Research in Rehabilitation and Social Integration (CIRRIS) at the Quebec Rehabilitation Institute and Department of Rehabilitation, Faculty of Medicine, Laval University, Quebec, Canada

**Keywords:** Balance deficits, Cerebrovascular accident, Gait, Gait retraining, Game-based rehabilitation, Physiotherapy, Posture, Rehabilitation, Stroke, Virtual reality

## Abstract

Rehabilitation interventions involving virtual reality (VR) technology have been developed for the promotion of functional independence post stroke. A scoping review was performed to examine the efficacy of VR-based interventions on balance and mobility disorders post stroke. Twenty-four articles in the English language examining VR game-based interventions and outcomes directed at balance and mobility disorders were included. Various VR systems (customized and commercially available) were used as rehabilitation tools. Outcome measures included laboratory and clinical measures of balance and gait. Outcome measures of dynamic balance showed significant improvements following VR-based interventions as compared to other interventions. Further, it was observed that VR-based intervention may have favorable effects in improving walking speed and the ability to deal with environmental challenges, which may also facilitate independent community ambulation. VR-based therapy thus has the potential to be a useful tool for balance and gait training for stroke rehabilitation. Utilization of motor learning principles related to task-related training may have been an important factor leading to positive results. Other principles such as repetition, feedback etc. were used in studies but were not explored explicitly and may need to be investigated to further improve the strength of results. Lastly, robust study designs with appropriate attention towards the intensity and dose-response aspects of VR training, clear study objectives and suitable outcomes would further aid in determining evidence-based efficacy for VR game-based interventions in the future.

## Introduction

Although the length of in-hospital stay following an episode of stroke has consistently decreased [1-3], many individuals return home with residual deficits. Balance and gait deficits are commonly observed in this population, leading to reduced ambulatory activity [4], limitations in activities of daily living and community participation [5,6], physical inactivity and subsequent deterioration in quality of life [7,8]. Therefore, rehabilitation efforts geared towards improving balance and mobility are important to facilitate functional independence and optimize community ambulation and participation. One of the promising intervention tools that is sought to facilitate this goal is virtual reality (VR) technology.

VR consists of a range of technologies that can be used to artificially generate sensory information in the form of a virtual environment (VE) that is interactive and perceived as similar to the real world [9,10]. Since VEs are interactive and game-like, they encourage active exploration, enhance engagement and provide motivation and enjoyment, thus allowing longer exercise sessions and improved treatment adherence [11-13]. VEs can be designed to resemble real-life scenarios including those encountered in the community [9,14]. It is not feasible to physically replicate realistic, community scenarios in the clinic or to safely train patients in the community. VR thus affords therapists with the unique opportunity to expose and train patients in these scenarios in a risk-free, graded manner, while providing intensive training and multi-sensory feedback [15,16]. These and other factors make VR-based intervention a useful adjunct or alternative to conventional therapy in re-training balance and gait dysfunctions post stroke. A review of the literature to explore the effect of VR-based interventions in re-training balance and gait and promoting independent community ambulation in this population is therefore important.

Several systematic reviews [17,18], meta-analyses [19,20] and a Cochrane review [21] have been undertaken to review the utility of VR technologies in retraining post-stroke individuals. Most of these reviews (with one exception [20]) had broader scopes of investigation and included upper limb retraining and/or cognitive rehabilitation. Further, these reviews considered only stronger study designs such as randomized controlled trials (RCT) for inclusion and thereby overlooked studies with different designs. We were, however, interested in examining the evidence on VR interventions on a targeted area (balance and gait post-stroke), with a broader and more flexible inclusion criteria as allowed in scoping reviews [22]. This allowed us to explore the added evidence to identify aspects of VR-based intervention that may prove useful in the treatment of balance and gait dysfunctions post-stroke. Further, we were interested in exploring with this scoping review, the utility of VR-based interventions in enhancing abilities required for community ambulation.

Community ambulation entails independent mobility outside the home [6] and involves dealing with environmental challenges such as low light, uneven terrain, external physical load, traffic, obstacles, time constraints etc. [23]. Various studies define diverse criteria for successful community ambulation [24]. For this review, we used one of the following criteria to identify results predictive of independent community ambulation:

1) post training gait speed ≥ 0.8 m/s, 2) functional ambulation category (FAC) of 5 (independent community ambulator) [25], 3) gait outcomes recorded in the community and, 4) outcomes related to negotiation of the environmental challenges (such as slope walking, obstacle negotiation etc.) [23].

The objectives of this scoping review were, therefore, to appraise the current state of information about the effects of VR intervention on balance and gait in post-stroke individuals and to explore the utility of VR-based interventions in facilitating independent community ambulation. The scoping review was conducted using the framework of Arksey and O’Malley [22], described in greater detail by Levac et al. [26].

## Review

### Search strategy

OVID MEDLINE, OVID EMBASE, PUBMED and PSYCINFO databases were searched using the terms “rehabilitation”, “virtual reality”, “stroke”, “balance”, “gait” etc. between the periods January 1950 to December 2013. The search strategies used in Ovid Medline and Pubmed databases are illustrated in Table 1. Similar strategies were employed for the other databases. Furthermore, cross references obtained from the included articles were also considered.

**Table 1:** **Search strategies**

**Database**	**Search strategy**	**Articles retrieved**
Ovid Medline	Rehabilitation. mp. or Rehabilitation AND Virtual reality.mp.	485
Ovid Medline	Virtual reality.mp. AND Stroke/OR postural balance/OR balance training .mp. OR Hemiplegia	166
Ovid Medline	Stroke.mp. or cerebrovascular accident/AND balance training.mp. or posture/ AND virtual reality.mp. or user-computer interface	13
Ovid Medline	Gait.mp. or Gait/or Gait disorders, Neurologic/Stroke.mp. or “National Institute of Neurological Disorders and Stroke”/or Stroke/User-computer interface/or virtual reality.mp.	50
Pubmed	“Stroke” [Mesh] OR “National Institute of Neurological Disorders and Stroke” [Mesh] AND (“gait”[Mesh] OR “gait disorders, neurologic” [Mesh]) AND (virtual reality)	133
Pubmed	“Rehabilitation [Mesh] OR “rehabilitation” [Subheading] AND (virtual reality)	565

### Selection of articles for review

1696 retrieved articles were first screened for relevance based on their titles and abstracts. Studies that were not published in the English language, available as abstracts only, that did not include post-stroke individuals or included a mixed etiology sample without separate description of outcomes related to the stroke sample, were excluded. Studies that - 1) used VR as a training tool for balance and mobility, 2) reported at least one outcome related to gait or balance and 3) published in the English language were included.

The included articles were scrutinised to extract information about VR systems, the training paradigms, outcomes and results. Twenty-four studies that met the inclusion criteria were retained for this scoping review. The subsequent sub-sections provide a synopsis of the study design, virtual environments (VE), outcome measures and the findings.

### Study designs

Of the included studies, eleven were small RCTs (Level II evidence according to CEBM levels of evidence [27]) [28-38], four were controlled trials with concurrent control group (Level III evidence) [39-42], two studies had no [43] or a historical control group (Level IV evidence) [44] and six studies presented non-empirical evidence consisting of case reports, case series or proof-of-principle studies (Level V evidence) [10,45-50]. The proportion of RCTs has shown a consistent rise over the years, suggesting an increase in methodological rigor of studies in the field.

### Participant characteristics

Some variability with respect to subject characteristics was observed among the included studies. Fifteen studies [28-35,38-40,42,45,47,49,50] recruited subjects with chronic stroke, seven [10,36,41,43,44,48] with sub-acute to chronic stroke, one [37] with acute stroke and one study did not provide information stroke chronicity [46]. Most studies included individuals between the ages of 50 and 80 years. Further, four studies [34-37] recruited in-patients receiving rehabilitation, twelve [10,28,30,38-40,42,45-48,50] recruited community-dwelling participants while seven studies [29,31,32,41,43,44,49] did not provide these details. A typical sample in most studies was thus middle-aged and old, community-dwelling chronic stroke survivors.

### VR systems

Both custom-made and commercially available VR systems were used in studies (Table 2). Custom-made systems were usually laboratory specific and often combined other devices with the VR interface such as robotic devices [31,33,45] treadmills [10,30,34,40,43] and others. Among commercially available systems, two studies used the Interactive Rehabilitation and Exercise system, developed to comply with specific rehabilitation requirements and customization according to the patient’s needs [29,32]. Seven studies used over the counter gaming consoles such as the Nintendo Wii™, Sony Playstation™ or the Kinect™ systems [35,37-39,41,42,47]. These are designed for the general population but are being increasingly used for rehabilitation.

**Table 2:** **VR systems and training protocols**

**Study**	**VR system**	**Training protocol**
Deutsch et al. (2004) [45]	Rutgers Ankle – an airplane simulation task required that the subject control the position of the airplane by using his ankle through a series of targets and not contacting them. For experiment 2, haptic effects were introduced to the Stewart platform and a new simulation that required the subjects to navigate a boat was added.	Exp 1 – 4 one hour sessions Exp 2 – one hour, 3 times per week for 4 weeks
Jaffe et al. (2004) [28]	Subjects walked on a motorized treadmill at self-selected walking speeds and viewed the real time display of their legs via a helmet mounted display wherein stationary images of obstacles were introduced. The subjects were required to step over the obstacle (take sufficiently higher and larger steps) to complete the task.	Control group: stepping over real foam obstacles Experimental group: stepping over virtual obstacles 1 hour sessions, 6 sessions over 2 weeks
You et al. (2005) [29]	IREX, GestureTek- stepping up and down, Sharkbait and Snowboarding	Control group: no intervention Experimental group: VR games - 60 minutes per day, 5 times per week for 4 weeks
Betker et al. (2006) [46]	COP-controlled video-game based exercise games – Under Pressure, Memory Match and Tic-Tac-Toe. The system consisted of a pressure mat which mapped the COP movements as the subjects moved. This was synchronized to the game motion software so as to match the COP movements for game play. The users were supposed to move in the AP and the ML direction at different speeds in order to play the games.	Eight 45-min exercise sessions over 3 weeks, 3 sessions in 1^st^ and 2^nd^ week each and 2 in the 3^rd^ week.
Fung et al. (2006) [10]	VR-based locomotor system with a self-paced treadmill mounted on a 6-degree of freedom of motion platform. Subjects reacted in three VE’s with 3 levels of complexity. Each VE involved walking 39 m within a predetermined time constraint. Progression from one level to the next was permitted only after successful completion of 3 trials within the time constraint, as the level of complexity increased with environmental perturbations and presence of moving obstacles.	Both control and stroke subjects received the same intervention. Training frequency or length of single session not defined.
Flynn et. al. (2007) [47]	EyeToy Play Station 2 gaming system. Games played required dynamic balance, upper extremity ROM, speed, cognition, reaction time and accuracy.	Playing games on the gaming console in the standing position 20 one hour sessions over 4 ½ weeks.
Yang et. al. (2008) [30]	Motorized treadmill placed in front of three 239 cm wide screens Task consisted of walking in a community VE resembling community scenarios in Taipei and involved negotiating slopes, avoiding obstacles etc.	Control group: treadmill walking while performing various tasks, Experimental group: walking in the VE 20 min per session, 3 sessions per week for 3 weeks.
Dunning et. al. (2008) [48]	SEMG coupled VR system that required the subject to contract and relax the agonist and antagonist muscles of the ankle to complete a “paint” game.	One hour session, 3 times per week for 8 weeks – 30 min functional lower extremity strength training and 30 min sEMG VR training.
Mirelman et al. (2009 [31]; 2010) [33]	Rutgers Ankle Rehabilitation System - 6-degree of freedom Stewart platform force-feedback system that uses ankle movements to navigate through a VE displayed on a desktop computer as in Deutsch et al. (2004)	Experimental group: Ankle exercises in the form of VR games along with the robot. Control group: training only with the robot, same exercises as the experimental group without VR. One hour sessions, 3 times per week for 4 weeks
Kim et al. (2009) [32]	IREX VR system - Shark Bait, stepping up/down and snowboard games were used.	Control group – 40 min of conventional physical therapy (CPT) Experimental group - 40 min of CPT + 30 min of VR therapy, 4times/week for 4 weeks
Walker et al. (2010) [43]	BWSTT + VR system – Body-weight supported treadmill training combined with VR. Subjects walked on a Biodex Gait Trainer 2 treadmill with an overhead Biodex Unweighting system while a virtual street scene was projected on to a 51-inch television monitor connected to a desktop. The scene was synchronized to the subjects’ motion via an inertial orientation tracking device mounted on a cap worn by the subjects.	2-3 sessions per week for 4-6 weeks for a total of 12 sessions per subject. Each training session lasted for 10 min initially and the duration was increased for the later sessions as tolerated.
Shin et al. (2010) [39]	Sony Playstation 2 Eyetoy play gaming system. Games such as Goal Attack, Table Tennis, Homerun, Knockout and Bowling were used. All of the games had a component of dynamic balance, speed and reaction time training.	Control group: no intervention. Game exercise group: 60-min sessions, 3 times per week for 6 weeks.
Yang et al. (2011) [34]	VR Treadmill system consisting of 4 components: 1) a commercial treadmill modified to operate at speeds ranging from 0.1 to 1 mph, 2) a personal desktop computer with a liquid crystal display projector, 3) a VR training program resembling a video-game developed from commercial software (3DWeb, superspace) consisting of scenes that involved walking in a park along a pathway with eight left and right turns respectively, and indoor activities such as turning a light on/off and opening doors, 4) interactive switches located on each side of the treadmill – stepping switches to turn left/right and touch switch for hand motion tasks.	Both groups: treadmill training (duration 20 min), three times per week for 3 weeks + regular therapy sessions. Experimental group: walking on the treadmill in the VE while stepping on the switches to affect turns. Control group: level walking on the treadmill without VR
Cikaljo et al. (2012) [44]	Balance Trainer (BT; Medica Medizin-technik GmbH) standing frame fixed to the base with passive controllable springs that control the stiffness of the standing frame which can move within two-degrees of freedom (AP, ML and combinations of both). Virtual environment was built in the 3D rendering program and involved moving along a path (through AP and ML movements) towards a goal while avoiding collisions with obstacles in the path.	Experimental group: BT + VR – 20 min per session, 5 times per week for 3 weeks (2 weeks clinic + 1 week telerehabilitation) Control group: BT – 20 min per session, 5 times per week for 4 weeks.
Cho et al. (2012) [35]	Conventional 42-inch LCD TV connected to a balance board gaming system (Wii Fit Balance Board, Nintendo, Japan). Commercially available games on the Wii such as balance bubble, ski slalom, ski jump, soccer heading, table tiling and penguin slide were used for training.	Both groups: standard rehabilitation program. Experimental group: additional 30-minVR training thrice a week for 6 weeks.
Feasel et al. (2011) [49] and Lewek et al. (2012) [50]	Integrated Virtual Environment Rehabilitation Treadmill (IVERT) system consisting of an instrumented dual-belt treadmill coupled to an immersive VE. The treadmill utilized a control algorithm to drive the treadmill speed in congruence with the user’s gait speed (estimated from the ground reaction force). VE consisted of a park with rolling hills, trees, rocks and a trail lined with fence posts. The VE was displayed with the help of three short-throw projectors that were mounted in front of a three-panel display placed a distance of 1.2 m from the front and 0.9 m at the sides respectively. The combined image display had a 170° horizontal and 60° vertical field-of-view. Asymmetry in gait was portrayed as a curved path in the VE and users were encouraged to maintain a straight path during trials.	Feasel et al.: One session consisting of 20-40 min of treadmill walking. Lewek et al.: Two post-stroke individuals performed 18 sessions over 6 weeks consisting of 20 min walking with the IVERT system followed by 10-15 min of overground walking. Each session lasted for 60 min (patient 1) or 45 min (patient 2).
Jung et al. (2012) [40]	The set-up consisted of a treadmill and an HMD through the virtual scene was displayed. The virtual scene consisted of a park stroll. The HMD displayed a 100-inch screen and had built-in earphones. Further information about the virtual scene and synchronization of the treadmill with the VR was not provided.	Experimental group: Treadmill walking with VR 30 min/day, 5 days/week for 5 weeks. Control group: treadmill walking without VR, following the same schedule as for experimental group.
Kim et al. (2012) [41]	Nintendo Wii sports software to play games involving tennis and boxing. The Wii games were displayed on a 30-inch TV display placed at 60 cm above the ground atop which was mounted the motion sensor. The motion sensor communicated with a remote that was either held on or strapped to the unaffected hand of the stroke subjects. Both games required varied motion and acceleration patterns to succeed.	Both groups received general exercises (not specified) for 30 min and electrical stimulation to the tibialis anterior muscle on the paretic side for 15 min. The experimental group received additional VR training for 30 min/session, three times a week for 3 weeks.
Cho et al. (2013) [36]	The set-up utilized a treadmill, a laptop, projector and speakers. The laptop was used to project real-world video recordings (VRRW) on to the projector which consisted of 10-min recordings of a 400 m walk track, a rainy 400 m walking track, a 400 m walking track with obstacles, walk in the community during daytime and nighttime and walking on a trail. Each recording was repeated thrice during the 30 min training period.	Both groups: standard rehabilitation program, 80 min per day, 5 times a week for 6 weeks. Experimental group: VRRW training, 30 min a day, 3 times per week for 6 weeks. Control group: treadmill training without VR for similar duration.
Fritz et al. (2013) [38]	Nintendo Wii (Wii sports and Wii Fit) as well as Playstation (Eyetoy play and Kinect) gaming systems were used to train the experimental group. The Wii Fit and Kinect were marketed as physical activity whereas Wii sports and Eyetoy were marketed as ‘fun-based’ games. The patients were encouraged to operate both systems on their own.	Control group: no intervention VR group: 50- 1 hour sessions consisting of 25-30 min physical activity and 25-30 min fun-based games. Frequency: 4 days/week for 5 weeks.
Rajaratnam et al. (2013) [37]	Nintendo Wii-fit and the Kinect gaming system were used to train the experimental group. Games that required subjects to shift weight in standing (Wii Fit and Kinect) and sitting (Kinect only) were used for training.	Control group: 60 minutes of conventional rehabilitation per session for 15 sessions. Experimental group: 40 min of conventional rehabilitation + 20 min of VR training per session for 15 sessions.
Singh et al., (2013) [42]	Nintendo Wii Fit plus with Balance Board: Balance Bubble game Xbox 360 Kinect: Rally Ball game, individuals who scored a gold medal on this game progressed on to the Reflex Ridge game.	Control group: standard group exercise therapy 2 hour sessions 2 times per week for 6 weeks. Experimental group: 90 minutes of standard group exercise + 30 min of VR balance games, 15 min each on the WiiFit and Xbox Kinect, twice per week for 6 weeks.

### VR-based interventions

- VR tasks: The tasks used in the studies generally reflected the training objectives. For instance, studies aimed at improving balance utilized balance training tasks [32,35,37,38,41,46,47] while those aimed at improving gait utilized treadmill walking [10,28,30,36,40,43,49,50] or components of gait training such as ankle range of motion (ROM), strength [31,33,45] or appropriate activation/deactivation of ankle muscles [48].
- Training dosage: Most studies used training sessions lasting 40-60 min (Table 2). Some studies employed shorter (20 min) training sessions [30,34]. The training frequency varied from 2-5 times per week and total training duration lasted between 2-8 weeks. Consequently, the total VR intervention showed a wide variation ranging between 2 to 22 hours. Typical training doses comprised of sessions of 40-60 min duration, 3-5 times per week for 3-6 weeks.
- Feedback: Apart from the obvious intrinsic visual feedback perceived from the VE, additional intrinsic auditory, somatosensory or proprioceptive information were manipulated in some studies. For instance, Fung et al. [10] used a six-degree of freedom motion platform to simulate slopes in the VE to impart proprioceptive information congruent to walking on inclined surfaces, while Deutsch et al. [45] used haptic inputs to simulate turbulence or sensation of collision. This multisensory feedback could have acted as an important facilitator of intrinsic learning of the tasks, while enhancing engagement with the VE. Some studies also provided additional extrinsic feedback through knowledge of performance (KP) or knowledge of results (KR). Nine studies provided both KR and KP [10,29,31,32,38,43-46], three studies provided only KR [28,41,47], while others [30,34-37,40,42,48] did not provide or report on provision of KR/KP feedback. KP was provided either by the system [10,29,32,43,45,46] through graphs depicting an outcome or movement quality, or from verbal feedback (about movement quality, area of improvement etc.) by the therapists present during training [31,38,44]. KR was usually provided by the system as visual (e.g. success scores, placards) or auditory (e.g. cheering and other sounds) feedback.

Other motor learning principles such as motivation, variable practice, and attention through enhanced engagement were not addressed explicitly in most studies. [Table 2].

### Outcome measures

The outcome measures utilized in the studies reflected body function and activities domain of the International Classification of Function (ICF [51]) and comprised of both laboratory measures and clinical tests. Balance assessment included center of pressure (CoP) measurements (CoP sway, sway velocity etc.) during static (quiet standing) [32,34,35,46], and dynamic postural tasks [34,36] as well as clinical tests such as the Berg Balance Score (BBS) [32,35-38,43,44,47]. Gait related outcomes were commonly measured during overground walking (reflecting transfer of VR training to overground gait) and included gait speed [28,30-32,34,36,38,39,42-44,48-50], spatiotemporal gait parameters (stride length, step length, cadence etc.) [28,31,32,34,36,49,50] and kinematic as well as kinetic gait parameters [33,48]. Clinical tests such as Timed-Up and Go (TUG) test [35,36,38,42,44,46,47], the 6-minute walk test [28,31,38,39,42,47] and Functional Ambulation Category (FAC) [29,31] were also reported.

Two studies measured ambulation in the community environment. Yang et al. [30] used the community walk test (time taken to walk 400 m in a community environment) while Mirelman et al. [31] reported on community walking activity (number of steps/day, average daily distance walked, speed, cadence etc.) using the Patient Activity Monitor. In addition, few studies reported outcomes reflecting challenges encountered during community ambulation. For e.g. Jaffe et al. [28] reported the obstacle test (the longest obstacle successfully crossed among a range of obstacle heights and lengths) and Fung et al. [10] reported on meeting time constraints, slope walking and obstacle avoidance. The details of all the outcomes reported can be found in Table 3.

**Table 3:** **Summary of studies**

**Study**	**Design, level of evidence**	**Population N, age, duration post-stroke**	**Type of stroke**	**Outcome measures**	**Results**
Deutsch et al. (2004) [45]	Pilot study, V	Chronic stroke **Experiment 1** – 69-year old, 10 months post-stroke (n = 1) **Experiment 2** – 1-8 years post-stroke (n = 3), age not given	Exp 1- Right middle cerebral artery CVA Exp 2 – not specified	Exp 1) VR-based measures including power, torque, accuracy,strength and range of motion Exp 2) Walking speed; 6-minute walk test (6MWT); Berg Balance score (BBS); foot strength	Exp 1) One grade increase in the strength of ankle evertors. Increased accuracy on the VR simulations (from 58% to 88%). Exp 2 –Increase in strength increase in 2 to 4 muscle groups and increase in walking speed (80 ft/min to 95 ft/min).
Jaffe et al. (2004) [28]	Randomized controlled trial, II	Chronic stroke (3.8 ± 2.2 years post-stroke), 60.7 ± 2.3 years; virtual object group (n = 10); real object group (n = 10)	Not specified	Percentage improvement on: 1) Balance tests (natural stance, natural stance eyes closed, on toes, tandem stance, tandem stance eyes closed, left leg only, right leg only) 2) Walking tests (walking velocity, cadence and stride length at self-selected and fast walking) 3) Obstacle test 4) 6-minute walk test (6MWT)	1) Results related to the Balance tests are not reported. 2) Greater percent improvements in walking speed (20.5% vs. 12.2%) and stride length for fast walking with VR 3) Obstacle crossing showed greater percent improvements with virtual obstacle protocol. Greater than 95% retention in all key gait parameters at 2 week follow-up 4) 6MWT: No significant changes post-treatment observed in both groups.
You et al. (2005) [29]	Experimenter blind randomized study - RCT, II	Chronic stroke (VR group – n = 5, 54.6 ± 3.0 years, 18.2 ± 2.3 months post-stroke control group – n = 5, 54.6 ± 3.4 years, 19.4 ± 4.3 months post-stroke)	Thalamic hemorrhage (n =2) Corona radiata hemorrhage (n =3) Corona radiata infarct (n = 5)	1) fMRI – Laterality Index (LI) 2) Functional Ambulation Category (FAC) 3) Modified Motor Assessment Scale (MMAS)	1) Bilateral activity in the sensory-motor cortex as seen before treatment either disappeared or decreased on the ipsilateral side indicating cortical reorganization. 2) All patients in the VR group were able to achieve a change of at least one level on the FAC as opposed to the control group. 3) Significant changes in MMAS.
Betker et al. (2006) [46]	Single-subject design: case report, V	n = 3, case 1 – 20-year old case 2 – 58-year old post-stroke (onset not provided) case 3- 14-year old	Case 1- cerebellar tumour excision with ataxia Case 2 – cerebrovascular accident infarction Case 3 – closed TBI (case 1 and 3 will not be discussed in the paper)	1) Tasks that involved maintaining static and dynamic balance 2) CoP excursion and sway path 3) Number of falls	1) Successful completion of oscillating head rotations, trunk bending and trunk rotation tasks post-exercise as opposed to failure pre-exercise. 2) Variable findings on CoP excursion and CoP sway path with increase in excursion and sway path in some and decrease in other tasks. 3) 5 falls pre-exercise as opposed to 2 falls post-exercise
Fung et al. (2006) [10]	Feasibility study, V	Sub-acute stroke (n = 2, 49 & 61 years, 2 and 4.5 months post-stroke) Control – healthy elderly (n = 1, 64 years)	Not specified	Ability to complete the locomotor task within the time constraint and without any collisions with the virtual obstacles.	Both subjects as well as the control were able to increase the walking speed to complete level 1 in all VE’s. The subjects were also able to eventually complete level 2 despite a considerable reduction in gait speed while negotiating platform movements. Both subjects did not reach level 3 to avoid obstacles successfully. The precise increase or decrease in gait speed is not specified.
Flynn et al. (2007) [47]	Case report, V	Chronic stroke (n = 1, 17 months post-stroke, 76-year old)	Cerebro-vascular accident, area not specified	Only those concerned with balance and mobility are listed here: 1) BBS 2) Dynamic Gait Index (DGI) 3) Timed Up-and-Go (TUG) 4) 6MWT 5) Functional Reach test (FRT)	Only those concerned with balance and mobility are listed here: 1) BBS increased from 51/56 pretest to 54/56 post-test 2) DGI – 16 pre-test to 21post-test 3) TUG - 12.73 s pre to 11.68 s post 4) 6MWT–1282 ft pre to 1337 ft post 5) FRT 11.33 in pre to 11.50 in post
Yang et al. (2008) [30]	RCT, II	Chronic stroke: VR group (n = 11, 55.5 years; 5.9 years post-stroke) Control group (n = 9, 60.9 years, 6.1 years post-stroke)	Not specified	1) Walking speed 2) Community walking time 3) Activities-specific Balance Confidence scale (ABC) 4) Walking Ability Questionnaire (WAQ) score	1) Insignificant change in walking speed but a trend towards increase. 2) Significant improvement in community ambulation time (6.14 ± 5.53 s); 3) ABC scores (8.86 ± 10.10points); 4) WAQ scores (3.45 ± 5.11points); All gains retained at 1 month follow-up.
Dunning et al. (2008) [48]	Case report, V	Sub-acute stroke (n = 1, 51 years; 9 months post-stroke)	Ischaemic infarct in the posterior limb of the left internal capsule	1) Modified Emory Functional Ambulation Profile (mEFAP) 2) Lower extremity portion of the Fugl-Meyer (FM) scale 3) Gait velocity: self-selected & fast 4) Temporal distance gait parameters 5) Lower extremity joint kinematics and kinetics	1) mEFAP – 4.4 s (11.2%) decrease post-intervention 2) FM – 5 point (23.8%) increase with largest changes for obstacles (17.2%) and stairs (13.7%) 3) Gait velocity increase post-intervention -Slow – 0.29 m/s (27.7%) fast – 0.26 m/s (16.4%) 4) Temporal distance gait parameters such as step length, cadence, stance and swing times also showed minor improvements post-treatment. 5) Ankle plantarflexor moment during push off increased 29.5% and 13.3% for self-selected and fast walking speeds respectively Greater hip extension at push off, improved knee extension at heel strike and greater ankle plantarflexion at push-off.
Mirelman et al. (2009; 2010) [31;33]	Randomized controlled trial, II	Chronic stroke Robotic VR group (n = 9; 61.8 years, 37.7 months post-stroke), robotic group (n = 9; 61 years, 58.2 months post-stroke)	Not specified	1) 6MWT 2) Community-based walking as measured by the PAM (no. of steps/day, average daily distance walked, speed, cadence, walking strides, maximum walking speed, longest consecutive locomotion period in minutes and longest consecutive distance travelled). 3) Self selected walking speed (SSWS) 4) Joint kinetics – ankle moments during stance and pre-swing, knee flexor moment during stance and push-off, hip flexor moment at initial swing, power at the ankle, knee and hip joints 5) Joint kinematics – ROM of the ankle and hip joints during the gait cycle	1) 6MWT – 21% increase in the Robotic VR group; 0.5% increase in the robotic group 2) Community based ambulation – significant differences seen in distance walked, no. of steps per day, average speed and maximum speed. All changes were retained in the robotic VR group at 3 months follow –up. 3) SSWS –24% increase (from 0.65 to 0.81 m/s) in the VR group vs. 2% (0.67 – 0.68 m/s) in the NVR group. 4) Joint kinetics- Ankle moment (barefoot walking) – VR group (from 0.74 ± 0.24 Nm/kg to 0.90 ± 0.31 Nm/kg, 21%) vs. NVR group (0.68 ± 0.17 Nm/kg to 0.67 ± 0.08 Nm/kg, 1.5%). Ankle power (barefoot walking) – significant increase in the VR group (0.63 ± 0.28 W/kg to 0.91 ± 0.45 W/kg; 44%) as opposed to the NVR group (0.5 ± 0.27 W/kg to 0.52 ± 0.26 W/kg; 4%). Retention at follow-up was seen in the VR group. 5) Kinematics –Barefoot walking Ankle ROM – increase from 29.3 ± 7.4 degrees (19.5%) in the VR group and from 32.6 ± 13.4 to 36.7 ± 3.2 degrees (3.3%) in the NVR. Both changes were reported to be statistically significant. Knee ROM – significantly greater increases (stance-34%, swing – 15.7%) in the VR group as compared to the NVR group (stance – 7.2%, swing – 3.9%). Both ankle and knee ROM gains were preserved at follow-up. Onset of push-off – improved from 55% of gait cycle in both groups to 57.7% of the gait cycle in the VR group only.
Kim et al. (2009) [32]	RCT, II	Chronic stroke: Experimental group (n = 12; 52.4 years, 25.9 months post-stroke); Control group (n = 12; 52.4 years, 25.9 months post-stroke)	Intra-cranial hemorrhage in the thalamus, putamen, basal ganglia (n = 13) Infarcts in the deep cerebral white matter, basal ganglia, putamen and pons (n = 11)	1) BBS 2) Modified motor assessment scale 3) 10 m walk test 4) mean CoP sway area; sway path and maximal sway velocity; antero-posterior (AP) and medio-lateral (ML) sway angles 5) Temporal distance gait parameters: cadence, velocity, step time, stance time, swing time, single/double support time, step/stride length.	1,2,3) Significant improvement in scores 4) reduction in sway area and maximal CoP velocity; AP and ML sway angles increased 5) cadence, step time, step length showed significant increases
Walker et al. (2010) [43]	Pre-post design, IV	Sub-acute to chronic stroke (3 weeks to 1 year post-stroke) N = 6, 53.4 years (49-74 years)	Ischaemic stroke with left-side hemiparesis	1) FGA 2) BBS 3) Overground walking speed Also reported pre-post treadmill walking speed, treadmill walking duration and percentage body-weight supported	Significant increases in 1) FGA (30%); 2) BBS (10%); and 3) overground walking speed (38%, change of 0.19 m/s pre-post). Treadmill walking duration – pre -10 min, post- 19.83 min Treadmill walking speed – pre – 1.31mph, post – 1.7 mph Weight support (% Body weight) – pre- 31.67, post – 18.33.
Shin et al. (2010) [39]	Pre-post, III	Chronic stroke (Control group, n = 16, 60.7 ± 9.2 years, 71.5 ± 33.9 months post-stroke; Game exercise group, n = 16, 60.8 ± 7.5 years, 69.2 ± 36.4 months post-stroke)	Control group – 11 ischemic and 5 hemorrhagic stroke; Game exercise group – 10 ischemic and 6 hemorrhagic stroke	1) 10 m walk test 2) 6MWT	1) Significant improvements in gait speed (0.86 ± 0.32 m/s pre-training to 1.10 ± 0.34 m/s post-training) in the game-exercise group as compared to controls. 2) Significant improvements were also seen in distance walked in the 6MWT (225.87 ± 60.16 m pre-training to 268.79 ± 56.42 m post-training) and was significantly larger than that seen in control group.
Yang et al. (2011) [34]	Randomized controlled trial, II	Chronic stroke (control group, n = 7; 65.7 ± 5.9 years, 16.3 ± 10.4 months post-stroke Experimental group, n = 7, 56.3 ± 10.2, 17.0 ± 8.6 months post-stroke).	Not specified	1) Standing eyes open -Maximum CoP displacement in the ML (CoPML) & AP (CoPAP) directions, excursion (CoPE), sway area (CoPA), & bilateral limb-loading symmetrical index (SI). 2) Sit to stand (STS): those included above + CoP excursion for the paretic foot (CoPE/P). 3) Walking: Stance time of the paretic limb (ST/P), number of steps of the paretic limb & contact area of the paretic foot.	1) Standing eyes open: No statistically significant change in any parameters. A tendency towards increase in CoPML, CoPAP, CoPE, SI and CoPA in the experimental group. 2) STS: a significant improvement in SI and CoPE/P in the experimental group. 3) Walking: Significant increase in stance time of the paretic limb in both groups. Significant increase in the contact area of the paretic foot in the experimental group.
Cikaljo et al. (2012) [44]	Pre-post (follow-up only for the experimental group), IV	Sub-acute stroke (control group: n = 22, age: 61.0 ± 7.4 years) Experimental group: n = 6, 58.5 ± 12.1 years)	Not mentioned	1) BBS 2) TUG 3) 10 meter walk time 4) Single leg stance on affected (SAE) and unaffected (SAU) side. 5) Additional outcomes for the experimental group: VR performance time and number of collisions.	1, 2, 3, 4) No statistically significant between group differences on any clinical outcome. Both groups improved over time on the clinical tests. 5) The experimental group showed improved performance and decrease in the number of collisions post- intervention.
Cho et al. (2012) [35]	RCT, II	Chronic stroke (experimental group: n = 11, age: 65.3 ± 8.4 years, 12.54 ± 2.58 months post-stroke; control group: n =11, age: 63.3 ± 6.9 years, 12.6 ± 2.5 months post-stroke)	Not described	Static balance using postural sway velocity (PSV) in the AP and ML directions with eyes open (EO) and eyes closed (EC) 1) PSV – APEO, 2) PSV – MLEO, 3) PSV – APEC, 4) PSV-MLEC. Dynamic balance 5) BBS score, 6) TUG (s)	Static balance PSV measures (1,2,3 and 4) did not show statistically significant changes pre and post treatment in both experimental and control group. 5) BBS: BBS scores improved in both groups post treatment; greater increase in the experimental (VR) group. 6) TUG: A significant decrease in TUG times in both groups, decrease significantly larger in the experimental VR group.
Feasel et al. (2011) [49] & Lewek et al. (2012) [50]	Feasibility study; case report, V	Feasel et al. – Hemiparesis due to various reasons (n = 5). Of these 2 were chronic stroke; 32 and 48 months post-stroke respectively. Lewek et al. Chronic stroke (n = 2; 18 and 21 months post-stroke)	Feasel et al. - Not described Lewek et al.- Patient 1 – right internal carotid artery stroke Patient 2 – embolic stroke to the left middle cerebral artery	Feasel et al. 1) Gait velocity 2) Overground gait symmetry before and after training for stance time, single support time and step length at comfortable and fast walking speeds. 3) Patients’ comments about usability (ease of learning, what was easy or hard, what they liked and did not like about the experience) Lewek et al.: 1) Gait speed (comfortable (CGS) and fast walking (FGS)), 2) Step length asymmetry ratio, 3) Stance time asymmetry ratio	Feasel et al. 1) Gait velocity in the first 5 min of treadmill walking was comparable to overground walking 2) No significant difference in gait symmetry between the first and the last minute of walking. The ‘best minute’ of symmetry was significantly more symmetric than the first minute. No significant differences in average gait symmetry before and immediately after training. 3) Participants reported the task to be mentally taxing. However, positive comments about having visual feedback (walking in the VE) and having a visual goal (keeping the walking path straight) were received. Lewek et al.: 1) Patient 1 improved the CGS from 0.49 m/s (using a large-base quad cane) to 0.84 m/s post-training (without using a cane). This improvement was preserved over the follow-up. The FGS also improved from 0.56 m/s pre-training to 0.95 m/s post-training. Similar results were seen with patient 2, CGS improved from 1.02 m/s pre-training to 1.28 m/s post-training (retained on follow-up) while FGS improved from 1.71 to 1.88 m/s (retained on follow-up). 2) Step-length symmetry improved from 1.52 to 1.32 (with cane) and 1.18 (without cane) in patient 1. The positive effects were maintained on follow-up. Patient 2 did not demonstrate step-length asymmetry to begin with. 3) Stance-time asymmetry did not improve in Patient 1, but improved from 1.11 pre-training to 1.04 post-training. These effects were lost to some degree on follow-up.
Jung et al. (2012) [40]	Pre-post design, III	Chronic stroke (control group: n = 10, age 63.6 ± 5.1 years, 15.4 ± 4.7 months post-stroke; experimental group: n = 11, age 60.5 ± 8.6 years, 12.6 ± 3.3 months post-stroke	Control group: 5 ischemic, 5 hemorrhagic and 6 right, 4 left paretic stroke; Experimental group: 7 ischemic, 4 hemorrhagic and 5 right, 6 left paretic stroke	1) TUG (s) 2) ABC (%)	Both groups improved significantly on outcomes 1 and 2 (TUG change: control: -0.8 ± 0.7 s, stroke: -2.7 ± 1.9 s; ABC change: control: 4.3 ± 3.3%; stroke: 9.5 ± 6.0%). Improvements seen in the experimental group were significantly larger than the control group.
Kim et al. (2012) [41]	Pre-post design, III	Sub-acute to chronic stroke (control: n = 7, age 55.0 ± 13.0 years,12.9 ± 6.1 months post-stroke; experimental: n = 10, age 41.3 ± 6.6 years, 12.6 ± 7.1 months post-stroke	Control group – 3 hemorrhagic, 4 ischemic stroke, Experimental group – 4 hemorrhagic, 6 ischemic stroke	1) Postural assessment scale for stroke patients (PASS), 2) MMAS, 3) Functional Independence measure (FIM)	1 & 2) Both groups showed significant improvements on the PASS and MMAS. The experimental group improved significantly more than the control group. 3) No significant differences were found on the FIM scores both within and between groups.
Cho et al. (2013) [36]	RCT, II	Sub-acute to chronic stroke (control group: n = 7, age 65.1 ± 4.7 yrs; experimental group: n = 7, age 64.6 ± 4.4 yrs)	Control group: 5 ischemic, 2 hemorrhagic; Experimental group: 4 ischemic, 3 hemorrhagic	Walking balance: 1) BBS, 2) TUG (s), Temporal gait parameters: 3) Gait speed (cm/s), 4) Cadence (steps/min), Spatial gait parameters (paretic side): 5) Step length (cm), 6) Stride length (cm), 7) Single limb support (%)	Both groups showed significant improvements on all outcome measure post-test as compared to pre-test (1-7). The change seen post-test was significantly greater in experimental group as compared to the control group in BBS scores, TUG, gait speed and cadence (1-4).
Fritz et al. (2013) [38]	Randomized matched single blind design, III	Chronic community dwelling stroke (control group: n = 13, age 64.5 ± 10.1 years, 3.6 ± 3.2 years post-stroke; Experimental group: n = 15, age 67.6 ± 9.3 years, 2.5 ± 2.6 yrs post stroke)	Control group: 4 left, 9 right paretic stroke; Experimental group: 9 left, 6 right paretic stroke	1) FM 2) BBS 3) DGI 4) 6MWT 5) 3 m walk test 6) 3 m walk test – fast 7) Stroke Impact scale 8) TUG	No between or within group difference on any outcome (1-8). The effect sizes for the outcomes in the VR group were larger than those seen in the control group. These changes were preserved at the 3-month follow-up.
Rajaratnam et al. (2013) [37]	Randomized controlled trial, II	Acute stroke (control group: n = 9, 65.3 ± 9.6 yrs; Experimental group: n = 10, 58.7 ± 8.6 yrs)	Control group: 8 ischemic, 1 hemorrhagic; Experimental group: 8 ischemic, 2 hemorrhagic	1) FRT 2) TUG 3) BBS 4) COP sway 5) Modified Barthel Index (MBI)	Both the control and experimental group showed significant improvements in the TUG (2) and the MBI (5) post-intervention. There were significant improvements in FRT (1) post-intervention in only the experimental group.
Singh et al. (2013) [42]	Controlled pre-post design, III	Chronic community-dwelling stroke (Control group: n = 13, age: 67 ± 8.4 yrs; experimental group: n = 15, age: 65.4 ± 9.8 yrs).	Not specified	1) TUG 2) 30 s sit to stand test (30sSTS) 3) Timed 10 m walk 4) 6MWT 5) Overall balance score (OBS): RMS of the combined AP and ML sway 6) Barthel Index	Both the control and experimental group showed significant gains in TUG (1) and 30s STS (2) tests post-training. However, no difference between control and experimental groups were found post-training on any outcome.

### Effectiveness of VR-based interventions

Varying results were obtained in CoP measures comparing VR-based interventions with non-VR-based interventions. While no significant differences among VR-based and other interventions were found for CoP measures during static balance tasks, significant differences between interventions were noted in these measures during dynamic balance tasks. For example, Yang et al. [34] found a significant improvement in bilateral symmetry and CoP excursion under the paretic leg during the sit-to-stand task, while Kim et al. [32] reported a significant improvement in sway angles during a dynamic weight shift task only following a VR-based intervention. Similarly, for clinical measures of balance, five studies reported significant improvements in BBS scores [32,35,36,43,47] and two studies [37,47] reported an improvement on the Functional Reach Test following VR intervention as compared to other interventions.

Ten of the fourteen studies that reported gait speed found significant increases after VR-based intervention in comparison with other interventions. In addition, significant improvements in other spatiotemporal gait parameters such as cadence [32], step length [32,36,50], step time [32], stride length [36] and gait symmetry [50] as well as improvements in ankle, knee and hip ROM, and greater ankle moment and power were found following VR-based intervention [33,48] over other interventions. (Please see Table 3 for a detailed account of the results).

Further, we examined study outcomes using our criteria for independent community ambulation. Participants from four studies were able to achieve an average gait speed of ≥ 0.8 m/s following VR intervention [30,31,36,38]. Also, among participants who received VR-based intervention, 3/5 participants from You et al. [29] and 4/9 participants from Mirelman et al. [31] achieved an FAC of 5 placing them in the unlimited community ambulation category. In addition, when gait measures included community walking, Yang et al. [30] and Mirelman et al. [31] found significant increases in community ambulation time and community ambulation distance and speed respectively following VR intervention. Furthermore, Jaffe et al. [28] and Fung et al. [10] also found improvements in the ability to negotiate perturbations encountered in the community such as slopes and obstacles. An increase in gait speed of ≥ 0.8 m/s, improvement on FAC, improvement in community ambulation measures, as well as increased competency in dealing with environmental perturbations, could lead to independent community ambulation in some participants. These improvements were not seen following other interventions, suggesting an added advantage of VR-based interventions over other interventions in facilitating independent community ambulation.

One study [29] explored the effect of VR intervention on cortical re-organization post-stroke, wherein a significant shift from bilateral (pre-training) to ipsilesional (post-training) activation of the primary sensory-motor cortex during walking-like movements was found, suggesting that VR-based training can facilitate neuroplastic changes in the cortex.

## Discussion

This scoping review was undertaken to appraise the impact of VR intervention on balance and gait in people post-stroke. Since our review included studies published up to December 2013, we were able to include additional papers as compared to the recent reviews [18,19]. Findings from this review indicate that VR-based interventions have an added advantage over conventional interventions in the improvement of balance while performing functional tasks, as well as in the improvement of gait speed and the quality of gait. Preliminary results also suggest that VR-based interventions may prove advantageous in promoting independent community ambulation. Some of the factors (e.g., repetitive variable practice, enhanced engagement, motivation, added feedback etc.) associated with the VR systems and the training paradigms used could be responsible for this added benefit.

### VR systems

An accurate estimation of the VR system that yielded maximum gains could not be obtained from this review. Both customized and commercially available systems seemed to have equally beneficial effects over non-VR-based interventions. A recent review by Lohse et al. [19] also reported a similar view.

### VR-based interventions

Intensive, task specific, variable practice in enriched environments with extrinsic (additional) feedback facilitates motor learning [52]. All of the studies included in this review used some or all of these components during VR training. However, some variability in the utilization of learning principles was found.

- VR tasks: The congruence of the study objectives, VR tasks and outcomes was an important factor that influenced results. For instance, outcomes related to static balance were not responsive to VR-based interventions that used dynamic balance training. Similarly, studies that used standing VR tasks failed to achieve improvements on gait-related outcomes [38,42,44]. However, tasks that trained walking-like activities or specific components essential for gait (ROM, strength etc.) did transfer to improved walking [29,31,45]. Task-specificity thus seems to be an important variable but not the only consideration in utilizing VR-based interventions.
- Training dosage: Considerable variability was observed in total training durations that ranged from 2 to 22 hours. Although, a higher number of repetitions and longer training times are known to have beneficial effects [52], study outcomes did not seem to depend exclusively upon the training durations. In fact, a combination of task-related training and dosage may have influenced outcomes. This was also observed by Fluet et al. [18] in their recent review.
- Feedback: VR-based interventions are inherently designed to provide rich visual feedback through the VEs. Further addition of auditory, haptic or proprioceptive inputs not only enhances engagement with the VE but also provides enriched environments for practice that may facilitate learning and underlying neuroplasticity [52,53]. In addition, most studies included in this review provided extrinsic feedback in the form of KP or KR. This may be especially important for learning in the post-stroke population as their intrinsic motor learning abilities may be compromised by the stroke [54]. However, the effect of this feedback on learning abilities in post-stroke individuals was not explicitly explored or reported in any study. Therefore, although it could be assumed that extrinsic feedback may have facilitated the performance, it was difficult to gauge the extent to which it may have impacted results following VR training.

In addition to the above factors, increased motivation and engagement due to game-like nature of these interventions and variable practice provided by the interactive simulations may have also contributed to the added benefits of VR observed in this review. However, even these aspects were not adequately addressed in most studies. This review, therefore, cannot comprehensively address benefits of VR-based interventions pertaining to facilitation of motor learning and dosing parameters.

### VR-based interventions to promote community ambulation

As mentioned in the earlier sections, VR can be used to create scenarios simulating real-life situations including those that simulate community environments and its challenges. This provides therapists with a unique opportunity to train patients in community scenarios but in a risk-free, graded fashion. All of the studies in this review that used treadmill walking with VR utilized VEs simulating walking in the community such as in a park or a road intersection. However, only two studies used measures that assessed transfer of training to actual community ambulation, whereas most others utilized clinical measures like gait speed and FAC that could at best be considered relevant for community ambulation. Nevertheless, positive results indicating improved abilities to navigate in the community were observed suggesting that VR-based interventions could prove to be a useful tool to train independent community ambulation. Future studies should identify this unique advantage and explore utility of VR-based training in this area though use of robust study designs and appropriate outcomes.

## Conclusion

Evidence from this scoping review suggests that VR-based interventions have the potential to become an effective tool in the treatment of balance and gait deficits post stroke. However, robust study designs that identify specific objectives and choose congruent and appropriate training tasks and outcome measures need to be employed in the future to ascertain appropriate intervention and dosing parameters and achieve optimal training of balance and gait in the post-stroke population.

## Abbreviations

VR: Virtual reality
VE: Virtual environment
RCT: Randomized controlled trial
TUG: Timed Up and Go
BBS: Berg balance score
CoP: Center of pressure
FAC: Functional ambulation category
ROM: Range of motion
